# Retrospective Case Series of Traumatic Brain Injury and Post-Traumatic Stress Disorder Treated with Hyperbaric Oxygen Therapy

**DOI:** 10.1177/0963689719853232

**Published:** 2019-05-28

**Authors:** R. Douglas Shytle, David J. Eve, Soel-Hee Kim, Allan Spiegel, Paul R. Sanberg, Cesar V. Borlongan

**Affiliations:** 1 Center of Excellence for Aging and Brain Repair, Department of Neurosurgery and Brain Repair, Morsani College of Medicine, University of South Florida, Tampa, USA; 2 Neurological Solutions, Inc., Palm Harbor, USA

**Keywords:** traumatic brain injury, post traumatic stress disorder, war veterans, hyperbaric oxygen therapy

## Abstract

Returning veterans are frequently diagnosed with traumatic brain injury (TBI) and post-traumatic stress disorder (PTSD). Considering a recent case-controlled study of hyperbaric oxygen therapy (HBOT) reporting a reduction in suicidal ideation, we investigated retrospectively three veterans with chronic TBI/PTSD symptoms who were treated with multiple rounds of HBOT with neurophysiological testing performed before and after treatment. Improvements were detected on parameters within neurocognitive domains, including reductions in suicide-related symptoms. These findings independently confirm that HBOT may be effective in treating specific symptoms of TBI/PTSD that are not currently addressed with existing therapeutic approaches.

## Introduction

Returning veterans frequently exhibit symptoms suggestive of mild traumatic brain injury (mTBI) even though they have not been diagnosed with a TBI^1^. The acute damage frequently results in chronic psychological and neurological symptoms that can be difficult to treat, including headaches, memory and attention deficits, and mood swings. Post-traumatic stress disorder (PTSD), an anxiety disorder caused by a stressful event and the subsequent memories of it, has been diagnosed in a proportion of TBI patients. Symptoms such as fatigue, irritability, difficulty concentrating, sleep disturbances, and depression are frequently observed in both TBI and PTSD patients, thus adding to the debate as to whether TBI/PTSD patients are suffering from true PTSD or whether they are exhibiting PTSD-like symptom as part of TBI sequelae^2–6^.

TBI is common both within the general population and also within the active military, particularly due to their exposure to improvised explosive devices (IEDs). Current treatments are not ideal and include off-label use of Food and Drug Administration (FDA) black box labeled psychoactive medications, which may even contribute to the high rates of suicidality among this population^7^. Therefore, there is considerable interest in discovering new therapies, particularly if they have the added benefit of reducing suicidality.

Hyperbaric oxygen therapy (HBOT) increases the presence of oxygen within the bloodstream and tissues, thus enhancing the oxygen content in areas of low perfusion, and potentially restoring oxygen to deprived regions following injury. Potential benefits of HBOT that have been demonstrated in animal models and could impact TBI and PTSD symptoms include stem cell migration, reduced inflammation, alterations in cerebral blood flow, and increased angiogenesis and neurogenesis (reviewed in Ding et al and Liu et al^8,9^).

The clinical data on HBOT for TBI is mixed, with clinical data in poorly constructed clinical trials suggesting benefit (see Weaver et al^10^ for review). However, a recent case control study of veterans of the US armed forces with mTBI or persistent post-concussion syndrome (PPCS), with or without PTSD, found significant improvements in PPCS and PTSD symptoms, neurological exam, memory, intelligence quotient, attention, cognition, depression, anxiety, quality of life, and brain blood flow following HBOT^11^. Simultaneously and most importantly, subjects experienced a significant reduction in suicidal ideation and anxiety—possibly the most significant finding in the study given the current veteran suicide epidemic.

This retrospective case series intended to examine whether improvements in neuropsychiatric, neuropsychological and other measures were apparent in three patients with chronic TBI/PTSD following HBOT.

## Case Series

The case files of Allen Spiegel, a practicing neurologist in Palm Harbor, FL, who has successfully used HBOT to treat numerous neurological conditions, were reviewed for military personnel patients that had previously been diagnosed with TBI/PTSD and were treated with HBOT. Patient charts were examined from two male patients and one female patient who were chronic sufferers of TBI/PTSD and had undergone computer-assisted assessments before and after receiving at least 20 HBOT at 1.5 atmosphere absolute (ATA; or higher). One patient also underwent a computerized mood assessment (CNS Vital Signs, Inc.) and one underwent neuropsychiatric testing (NeuroPsych™^12^). The patients did not pay for the treatments as they were funded by the Healing Heroes Network, a 501(3)c nonprofit organization of which Dr. Spiegel is the Medical Director. All three patients expressed interest in receiving HBOT, and the risks versus benefits of HBOT were discussed at length prior to their consent for treatment. None of the patients had a prior history of pneumothorax, congestive heart failure, emphysema, diabetes, or sinus disease that would preclude the use of HBOT. All personal identifying information was redacted to protect the identity of the patients and this retrospective case series study was approved by the USF IRB. The details for HBOT are described in Table 1 for each patient.

**Table 1.** HBOT for patients 1–3.

Patient	Treatment
1	20 HBOTs in 29 calendar days at 1.75 ATA once/day, approximately 5 days/week
2	30 HBOTs in 30 calendar days at 1.5 ATA twice/day, approximately 5 days/week
3	a) 25 HBOTs in 22 calendar days at 1.5 ATA, twice/day, approximately 5 days/week b) 10 HBOTs at 1.5 ATA, once daily, approximately 5 days/week

HBOT was performed in a monoplace chamber with 100% oxygen and each session was 60 min total dive time.

### Case 1

Case 1 is a 29-year-old female who was exposed to daily IED blasts and numerous burn pit exposures while transporting materials from one place to another in her role as a US Army truck driver during a 1 year tour of active duty in Iraq.

Upon returning to her home base in Europe, she began to experience numerous cognitive deficits, including memory impairment, exemplified by not remembering people such as neighbors, or how to get to places that she once knew quite well, and would frequently get lost. She also had difficulty recalling computer passwords. Once back in the US, she reported having little recall of her life prior to being in Iraq. She had problems concentrating and remembering things she had read, along with poor recall of skills that she was once formerly proficient in. In addition, she could not recall if she had paid her bills and had difficulty keeping appointments. These cognitive deficits were frequently associated with depression, traumatic flashbacks, severe headaches, eye twitching, head twitching, stuttering of speech, and muscle fatigue. She described having “cycles of PTSD” associated with difficulty sleeping with frequent awakenings throughout the night. She had also experienced episodes of lost consciousness during the day with one episode lasting 4 h. She reported having been exposed to hazardous chemicals, including radiation, while in Iraq, and having acquired numerous chemical allergies.

She was diagnosed by the VA as having mild TBI, PTSD, high anxiety, asthma, chemical allergies, chronic back pain, gastroesophageal reflux disease (GERD), irritable bowel syndrome (IBS), tinnitus, fibromyalgia, rheumatoid arthritis, and possibly schizophrenia. She was placed on numerous medications by the VA, including Seroquel, Effexor, Lexapro, ranitidine, gabapentin, Xanax, and albuterol, all of which were poorly tolerated and eventually discontinued.

At the time of treatment, her medications included Trazodone, Valium, duloxetine, and other medications, the names of which she could not recall. She reported previously having smoked cigarettes, but had since quit, and denied the regular use of alcohol or other addictive drugs.

#### Initial assessment

While the patient was awake and alert, she appeared depressed, anxious, and tense. Otherwise, her physical and neurological examinations were unremarkable. Computerized mood and neuropsychological assessments corroborated self-reported emotional disturbances and cognitive deficits, with marked deficiencies in reaction time and verbal memory and below average deficits in executive function, composite memory, attention, and cognitive flexibility. The general clinical impression was notable for a 29-year-old female with symptoms consistent with TBI, PTSD, anxiety, and fibromyalgia.

#### Follow-up assessment

**Fig. 1:**
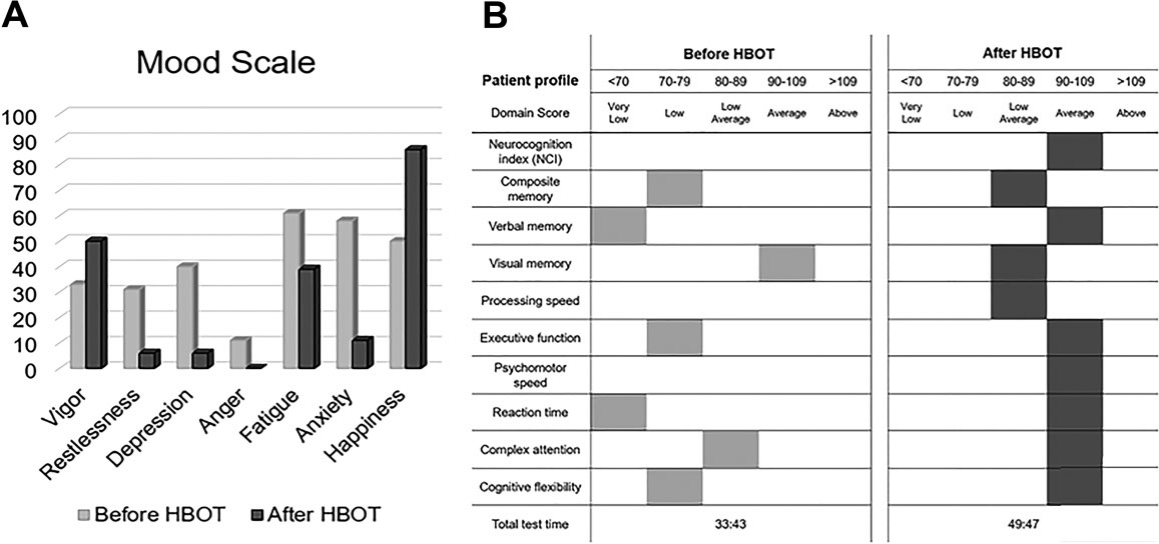
Computerized mood and neuropsychological assessments pre- and post-HBOT in female patient 1.

Overall, the patient reported significant improvements in both cognitive function and mood as well as significant reductions in PTSD symptoms, with only the rare occurrence of flashbacks. Specifically, she noted significant improvements in her judgment, memory, social interactions, reduced anxiety, and stress. Her frequent headaches and episodes of nausea also resolved. While her fibromyalgic pain had significantly diminished, she still noted lower back pain and wished to be considered for spinal decompression therapy. As shown in Fig 1 below, computerized mood and neuropsychological assessments corroborated self-reported improvements in mood and cognitive deficits, with a return to average scores in most neuropsychological domains.

#### Follow-up assessment (1 year later)

The patient reported that she had been doing well following HBOT, but unfortunately, she sensed that her condition had recently relapsed. She had been misplacing her possessions and could not recall the names of people she should know. She had lost her track of time, had difficulty in organizing herself, and had forgotten how to cook. She was under a fair amount of stress and anxiety and had started smoking cigarettes again. Otherwise, her physical and neurological examinations were unremarkable. The patient said that she would consider receiving more HBOTs, but no further follow-ups were found in her case file.

### Case 2

Case 2 is a 27-year-old male who sustained his first IED blast concussion with immediate loss of vision on his first night in Iraq. He subsequently sustained numerous IED blast exposures with associated episodes of dizziness, confusion, and headaches, but does not recall ever having lost consciousness. He was suffering from ongoing depression and alcoholism and was drinking as much as US $1000 worth of alcohol in 2 weeks. He had personally noted changes in emotion, decreased motivation, with diminished empathy and sympathy for others. He found that his way of life had changed significantly, and he did not feel accountable or responsible for things that he ordinarily should. There had been significant anxiety, stress, and tension with diminished libido and affection for his wife.

According to his wife, also present for the assessment, he was tense, anxious, had difficulty sleeping at night, and diminished motivation. Before his deployment, she reported that he was often considered the “life of the party”, but he had become more reclusive. For the first 6 weeks after returning to the US from his tour in Iraq, she said he slept with a knife by his side at night, and paced the perimeter of the house during the day. He had told her that he felt “dead inside” and had difficulty concentrating and comprehending what he had read while taking some college courses.

The patient had undergone numerous neurological assessments and had been seen by two other physicians whose findings both were consistent with TBI. Although no currently prescribed medications were noted, the patient reported drinking alcohol excessively and smoking three to four packs/week, but denied using any other addictive substances.

#### Initial assessment

The patient was awake and alert with appropriate affect. Otherwise, his physical and neurological examinations were unremarkable. Computerized neuropsychiatric and neuropsychological assessments corroborated self-reported mood disturbances and cognitive deficits. Severe levels of psychiatric symptoms were detected in domains of impulsivity, anxiety, fatigue, sleep, depression, and mood stability with moderate suicidality levels and mild aggression levels. Marked cognitive deficits were detected in executive function and processing speed, along with below average performance in complex attention and cognitive flexibility. The general clinical impression was notable for a 27-year-old male with symptoms consistent with TBI and PTSD.

#### Follow-up assessment

Other than some mild tinnitus in the right ear a few days earlier, the patient reported “doing well” with significantly reduced anxiety compared with before HBOT. He also reported sleeping better and appeared more motivated. It was notable that shortly after initiating HBOT he had started going to the gym regularly. He also reported gradual and progressive improvements in his memory, and he no longer felt depressed. Despite still “having a few beers, a few nights a week,” the patient appeared to show signs of therapeutic benefit from HBOT.

**Fig. 2:**
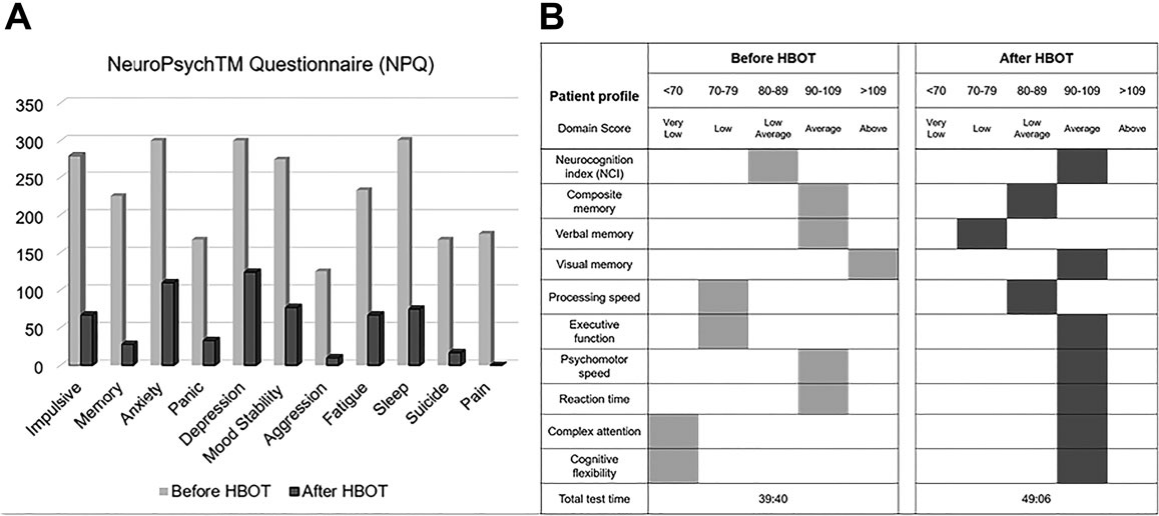
Computerized neuropsychiatric and neuropsychological assessments pre- and post-HBOT in male patient 2.

As shown in Fig 2, this clinical impression of significant therapeutic benefit was further corroborated by subsequent computerized neuropsychiatric and neuropsychological assessments, which revealed marked normalization of previously severe psychiatric symptoms, with only mild levels remaining for sleep disturbance, anxiety, depression, and mood instability. Likewise, the formerly severe cognitive deficits in executive function and processing speed, and below average performance in complex attention and cognitive flexibility, had completely resolved, with only a below average deficit in verbal memory remaining.

### Case 3

Case 3 was a 26-year-old male on active duty in Iraq 4 years earlier, when he sustained injuries from various IEDs, explosions, and rocket fire, which threw him against a concrete barrier, thus striking his head with subsequent loss of consciousness. Upon awakening, he had nausea, vomiting, dizziness, headache, and blood coming from his ear due to a ruptured ear drum. After 24–48 h observation he was sent back into battle. Subsequently, he had had 3–4 episodes in total of head trauma due to rocket propelled grenades (RPGs) and roadside bombs, one of which happened while serving in Afghanistan.

At the end of his tour of duty in Afghanistan, it was noted that he had some cognitive impairment, exemplified by difficulty recalling where he was going and what he was going to do. Repeatedly, people had to remind him what his duties encompassed for that day. He was medically discharged and had ongoing cognitive impairment, thus prompting evaluation for HBOT.

At the time of initial assessment, he complained of memory impairment exemplified by poor recall of recent events, difficulty recalling people’s names, misplacing possessions, and difficulty in recalling names of people he had met recently. Old friends, however, he recalled without difficulty. There was no day or night confusion, headaches, or change in personal hygiene. He had frequent flashbacks of his time in battle. He had lost interest in many of the things that he used to enjoy, including working out and going out, and now had a tendency to lock himself in a room preferring solitude, which was a major change in his personality. The patient had three to four migraine headaches per week, which he described as sharp, dull, pounding, and throbbing in nature and rated a 6–9/10 in severity. When the headaches occurred, they could last anywhere from 3 to 24 h and were frequently associated with nausea, vomiting, photophobia, and visual obscuration. The patient also complained of being more irritable, agitated, and requiring anger management at times. He complained of dizziness when his migraines were severe, but there had been no change in his sense of smell. He did admit to being depressed and extremely anxious. MRI’s of the brain performed in the past were notable for “clots,” but the report could not be located.

His current medications were buspirone, hydrochlorothiazide (HCTZ), Topamax, and Oxcarbazepine. The patient drank four alcoholic beverages a week, smoked one pack of cigarettes a day, and denied using any other addictive drugs. He is a right-handed gentleman who completed college.

#### Initial assessment

The patient was awake and alert with appropriate affect. Otherwise, his physical and neurological examinations were unremarkable. Computerized neuropsychiatric and neuropsychological assessments corroborated self-reported mood disturbances and cognitive deficits. Severe levels of psychiatric symptoms were detected in domains of impulsivity, anxiety, fatigue, sleep, depression, and mood stability. In addition, marked cognitive deficits were detected in composite and verbal memory, along with below average performance in verbal memory and reaction time. The general clinical impression was notable for a 26-year-old male with symptoms consistent with cerebral concussion, TBI, and PTSD.

#### Follow-up assessment

The patient stated that, over the past several days, he had been feeling excellent in regard to his previous symptoms, but on HBOT #25 he began to have an increase in anxiety, but was unclear as to why. He also said that when he went into the HBOT chamber, within 15 min he began having a cramping discomfort in his stomach necessitating its discontinuance. He believed that this may have been caused by food poisoning, so HBOT was postponed for the next 3 days and then resumed at 1.5 ATA/60 min total dive time, one/day, approximately 5 days/week until he had completed 35 HBOTs in total, at which time a computerized neuropsychological assessment was performed.

**Fig. 3:**
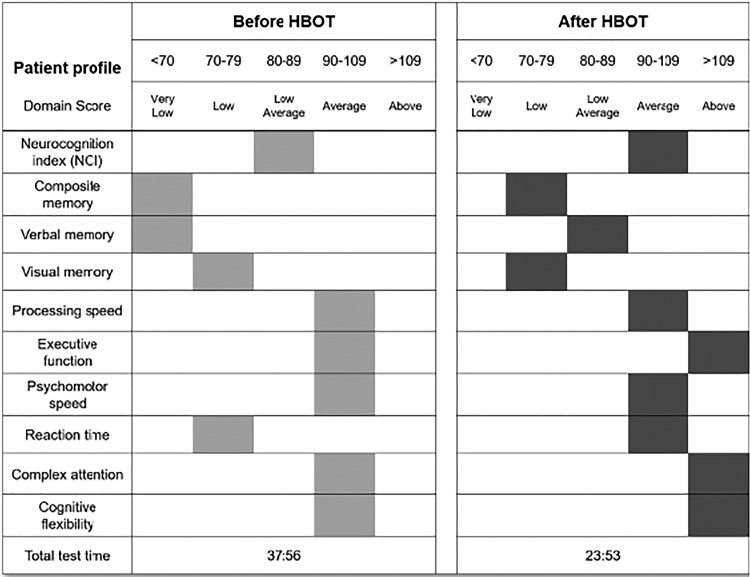
Computerized neuropsychological assessments pre- and post-HBOT in male patient 3.

As shown in Fig 3, some therapeutic benefit was quantified by subsequent computerized neuropsychological assessment, which revealed some improvement of formerly severe cognitive deficits in composite and verbal memory, and a return to average for the neurocognitive index. Interestingly, executive function, complex attention, and cognitive flexibility had moved from average to above average scores.

## Discussion

These three cases consisted of two males and one female, who, at the time of initial assessment, suffered from residual and disabling symptoms of TBI and PTSD primarily as a result of head injuries sustained years earlier while on active tours of duty in the US military. While the precise parameters of their ailments and treatment were not identical between patients, they all exhibited consistent improvement in overall cognitive function such as executive function, with only one patient showing a slight worsening in verbal memory following HBOT.

In addition, all three patients demonstrated different degrees of neuropsychiatric improvement following HBOT, two of which were further corroborated by independent computerized neuropsychiatric assessments, with one (case #2) showing nearly complete remission of suicidal symptoms. Overall, it appears that the patients seemed to be “happier” and more motivated following HBOT, which is consistent with the recent findings by Harch et al, reporting a significant reduction in suicidal ideation following HBOT^11^. Overall, these findings are also consistent with a previous double-blind placebo controlled study that found clinically significant reductions in symptoms of depression as measured by the Hamilton Depression Scale following HBOT during the convalescent stage in 60 patients following cerebral hemorrhage^13^.

While such retrospective case studies lack the experimental power of large sample sizes to draw the definitive conclusions required for regulatory and policy decisions, they do provide valuable qualitative insights for the practicing physician, which are not always possible from large prospective controlled trials. For example, one of the patients in this retrospective case study was an alcoholic with moderate levels of suicidality—symptoms which would have precluded him from participating in a standard controlled trial due to ethical and/or liability concerns of institutions or companies that typically fund such trials. While this patient apparently did not significantly reduce his level of drinking alcohol, his initial moderate levels of suicidality showed a complete remission following HBOT. Suicidality is a particularly lethal and underappreciated psychiatric symptom in this high risk population^14–16^.

Further research should be conducted to investigate potential mechanism(s) of action for HBOT’s putative anti-depressive and anti-suicidal effects. In this regard, altered stress reactivity is considered to be a risk factor for both major depressive disorder and suicidal behavior^17^. Recent studies found significantly reduced expression of spermidine/spermine N1-acetyltransferase 1 (SAT1), the rate-limiting enzyme involved in catabolism of the polyamines spermidine and spermine in the polyamine stress response (PSR), across multiple brain regions in depressed individuals who died by suicide^18^. Follow-up studies found that SAT1 transcription is influenced by lithium, and that this effect was altered in bipolar disorder patients who completed suicide, further supporting a role for polyamines in suicide^19^. Overall, these findings suggest that depression/suicide may be at least partially influenced by reduced expression of SAT1 resulting in low brain levels of putrescine and/or high levels of spermidine/spermine. Interestingly, exposure to HBO in mice was reported to significantly increase brain putrescine content with no significant changes in spermidine or spermine levels^20^. Thus, future studies should investigate the potential relationship between the anti-suicidal effects of HBOT and regulation of polyamine brain levels via SAT1 expression.

Future clinical trials should include comprehensive specific outcome measures of direct relevance to patients rather than the use of a more general overall disease measure to help reveal the benefits of a treatment. It is worth noting that the proposed Brain Injury and Mechanism of Action (BIMA) clinical trial (NCT01611194) has been designed to explore more comprehensive and exhaustive parameters that are relevant to TBI and PTSD patients^10,21,22^. Expanding the endpoint measures is further supported by Wolf et al^23^, who observed improvements in some individual components of the cognitive and PTSD testing performed following concussive subgroup analysis of the TBI patients in their randomized “control” clinical trial. With the FDA expectations of more thorough suicide assessments in clinical trials, the Sheehan-Suicidality Tracking Scale (S-STS) accommodates all the FDA suicide assessment expectations into a single assessment and would be useful in future HBOT studies in TBI and PTSD patients^24^.

The treatment for these patients was funded by the Healing Heroes Network. The cost of HBOT in a private clinic is US $10,000–20,000 for an adequate trial, meaning that the number of patients who can be treated is currently limited by financial constraints, which the Healing Heroes Network can alleviate for some patients. Further studies exploring the potential benefits of HBOT to treat specific facets of TBI/PTSD could lead to approval of this technique for additional funding, and thus reduce the burden on the patients. Only time and additional studies will tell which facets of TBI/PTSD are improved by HBOT and whether the cost-benefit ratio makes HBOT a worthwhile therapy.

Long-term follow-up will be helpful in determining whether the neuropsychological and neuropsychiatric benefits (and potential deficits) of HBOT are maintained or are only short term. It is possible that the apparent deficit in visual memory (one of three patients) was related to disease progression rather than HBOT, though with the absence of a placebo group, this is difficult to ascertain. If there appears to be only a short term benefit, then additional rounds of HBOT could be investigated to determine whether long term effects are observed. However, the exploration of treatment effects on specific neuropsychological and psychiatric parameters rather than the overall TBI/PTSD disorder does reveal more promising results.

Obviously, there are important limitations to any case series. The small sample size (*n* = 3) makes it difficult to make any definitive conclusions. This is further compounded by the fact that different parameters were measured and different time frames of treatments were administered as well as different follow up times. Other biological and physiological measures were also not available and follow-up information was limited. However, this case series provides anecdotal evidence that while HBOT may not modify all neuropsychological and psychiatric parameters of TBI/PTSD patients, improvements are observed in some measured parameters associated with suicidality, which serve to bolster the recent findings by Harch et al. (2017)^11^ and thus warrant further investigation to determine the nature of possible anti-suicidal properties of HBOT in future clinical trials. If HBOT can alleviate the suffering of a subset of such veterans, it represents a relatively safe and immediately accessible treatment that requires very little additional development.
